# Assessing Effects of Salinity on the Performance of a Low-Cost Wireless Soil Water Sensor

**DOI:** 10.3390/s20247041

**Published:** 2020-12-09

**Authors:** Srinivasa Rao Peddinti, Jan W. Hopmans, Majdi Abou Najm, Isaya Kisekka

**Affiliations:** 1 Department of Land, Air & Water Resources, University of California Davis, Davis, CA 95616, USA; speddinti@ucdavis.edu (S.R.P.); jwhopmans@ucdavis.edu (J.W.H.); mabounajm@ucdavis.edu (M.A.N.); 2 Department of Biological and Agricultural Engineering, University of California Davis, Davis, CA 95616, USA

**Keywords:** soil water sensor, capacitance sensor, laboratory calibration, wireless sensor, soil water content

## Abstract

Low-cost, accurate soil water sensors combined with wireless communication in an internet of things (IoT) framework can be harnessed to enhance the benefits of precision irrigation. However, the accuracy of low-cost sensors (e.g., based on resistivity or capacitance) can be affected by many factors, including salinity, temperature, and soil structure. Recent developments in wireless sensor networks offer new possibilities for field-scale monitoring of soil water content (SWC) at high spatiotemporal scales, but to install many sensors in the network, the cost of the sensors must be low, and the mechanism of operation needs to be robust, simple, and consume low energy for the technology to be practically relevant. This study evaluated the performance of a resistivity–capacitance-based wireless sensor (Sensoterra BV, 1018LE Amsterdam, Netherlands) under different salinity levels, temperature, and soil types in a laboratory. The sensors were evaluated in glass beads, Oso Flaco sand, Columbia loam, and Yolo clay loam soils. A nonlinear relationship was exhibited between the sensor measured resistance (Ω) and volumetric soil water content (*θ*). The Ω–θ relationship differed by soil type and was affected by soil solution salinity. The sensor was extremely sensitive at higher water contents with high uncertainty, and insensitive at low soil water content accompanied by low uncertainty. The soil solution salinity effects on the Ω–θ relationship were found to be reduced from sand to sandy loam to clay loam. In clay soils, surface electrical conductivity (*EC_s_*) of soil particles had a more dominant effect on sensor performance compared to the effect of solution electrical conductivity (*EC_w_*). The effect of temperature on sensor performance was minimal, but sensor-to-sensor variability was substantial. The relationship between bulk electrical conductivity (*EC_b_*) and volumetric soil water content was also characterized in this study. The results of this study reveal that if the sensor is properly calibrated, this low-cost wireless soil water sensor has the potential of improving soil water monitoring for precision irrigation and other applications at high spatiotemporal scales, due to the ease of integration into IoT frameworks.

## 1. Introduction

Soil water content (SWC) is a key variable for driving plant growth, precision irrigation management [1,2], and coupled hydrological, environmental, climatological, and ecohydrological processes [3]. SWC plays a significant role in hydrology to understand the hydrological cycle [4], rainfall patterns [5], interflow [6], and groundwater recharge [7]. The proper estimation of SWC is essential in computing actual evapotranspiration using the soil water balance [8], food security research [9], carbon balances [10], pollution detection [11], and hydrologic modeling [4]. Thus, the determination of spatiotemporal variability of SWC is essential for many applications, and several measurement techniques have been developed over the last several decades. Soil water content is measured as volumetric water content (VWC). Most of the soil moisture sensors infer VWC based on changes in thermal properties or electrical properties of the soil [12,13]. The electrical-based sensors used to measure VWC are based on the propagation of electromagnetic (EM) waves in the soil. These sensors fall into various types, e.g., time-domain reflectometry (TDR), frequency-domain reflectometry (FDR), capacitance, and resistance sensors [14].

There are several techniques available to monitor SWC content at the point and larger spatial scales [15,16]. The gravimetric method is the only direct, and most accurate, method to estimate SWC at the point scale, but it is destructive, laborious, and does not allow for real-time monitoring [17]. In recent years, electrical and EM methods have given more emphasis to characterizing soil electrical properties and SWC monitoring in space and time [18,19]. Ground-penetrating radar (GPR) and electrical resistivity tomography (ERT) methods have been developed to map the spatial variability of SWC at the plant to the field scale [20]. But, as these methods are not amenable for automation and use empirical models to estimate SWC, there is a need for advanced techniques that can assess field- to catchment-scale soil moisture with high spatiotemporal resolution [5].

Farmers face reduced agricultural productivity when there is a lack of water for irrigation, e.g., during droughts or in the presence of regulations that constrain water supplies. Using more sensors to monitor SWC at the farm level is expensive and return on investment may take a long time. Therefore, it is necessary to create a cost-effective and user-friendly system for monitoring soil water [21]. Recently wireless sensor networks (WSNs) have been developed to serve various purposes, including irrigation management, observation of ecohydrological processes, validation of remote sensing soil moisture products, and spatial characterization of soil properties [22]. To include a large number of sensors in the network, the sensor must be low-cost with a seamless wireless communication protocol. Besides, the sensors require soil-specific calibration in the field as well as in the laboratory. Further, the sensors typically show significant sensor-to-sensor variability, which can affect the measurement accuracy [21,23,24] if not individually calibrated. Over the last several decades, numerous calibrations were performed on various capacitance, TDR, and FDR soil moisture sensors, using various fluids, soils, and different statistical curve fitting methods.

Capacitance soil moisture sensors operate on the dielectric principle, which essentially consists of a pair of electrodes that form a soil capacitor acting as a dielectric in between. There are several ways to measure the capacitance, including using the capacitor reactance to form a voltage divider, constructing an RC oscillator where the capacitance calculates the frequency, or measuring the capacitance through charging the capacitor and discerning the charging time. When the battery is attached to a series resistor and capacitor, the initial current is high as the battery carries charge from one plate of the capacitor to the other. The transient behavior of the battery-powered circuit, the resistor, and the capacitor is regulated by the Ohm law, the voltage laws, and the capacitance concept. Mathematically, the relationship can be expressed as:

(1) $$V_{battery}=V_{resistance}+V_{capacitance}$$

According to Ohm’s law and definition of capacitance, Equation (1) can be written as:

(2) $$V_{battery}=IR+\frac{Q}{C}$$

where *R* is the resistance in the circuit, *Q* is a charge, and *C* is the capacitance.

**Figure 1:**
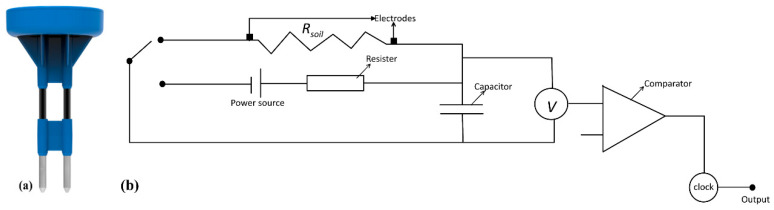
(**a**) The Sensoterra B.V. soil moisture sensor and (**b**) the circuit diagram of the sensor with resister, capacitor, power source, clock, and comparator.

Sensoterra B.V. has recently developed a robust, easy-to-use, and fully wireless sensor for measuring soil moisture [25] in different soil types. These sensors measure SWC based on the principle of resistance–capacitance (RC oscillator) [25]. The electronic circuit (Figure 1b) allows the current to flow through the ground during the sensing process. Due to direct current (DC), the circuit does not require high-frequency converters, and the installation in hard ground, or at relatively deeper depths, is simple [25]. By enabling the loop between the power source and DC current, the DC source can be charged for a predetermined time period (e.g., 10 ms), with the current flowing from the source through the charging resistor [25]. Assuming that the resistance is known, this results in a predetermined charging at the initial level, which can be expressed mathematically as Equation (3):

(3) V=I(t) R+q(t)C

where I(t) is the current, R is the resistance of the resister, q(t) is charge on the capacitor, and C is the capacitance of the capacitor. For continuously varying charge, the current I(t) is defined by a derivative $\frac{dq}{dt}$, and by using the expression in Equation (2), Equation (3) can be rewritten as:

(4) $$V=\frac{dq\left(t\right)}{d\left(t\right)}\;R+\;\frac{q\left(t\right)}{C}$$

Assuming the charge to be zero at the start, Equation (4) can be solved as:

(5) $$q\left(t\right)=VC\left(1-e^{\frac{-t}{RC}}\right)$$

For a long duration (*t* >> *RC*), the second term in the above Equation can be neglected, and thus the charge (q) would become VC. The power sources are no longer present while discharging the capacitor. Hence, Equation (4) can be written as:

(6) $$0=\frac{dq\left(t\right)}{d\left(t\right)}\;R_{soil}+\frac{q\left(t\right)}{C}$$

Assuming that the impedance is mainly resistive, for discharging the capacitor from a fully charged state through the current path, the solution for the above equation can be written as:

(7) $$q\left(t\right)=VC\left(e^{\frac{-t}{R_{soil}C}}\right)$$

where $R_{soil}$ is the ground impedance resistance component. Consequently, the only unknown entity in Equation (7) is resistance, when the time required to discharge the capacitor from the initial level to the predetermined level is measured. Hence, by using the capacitance from two electrodes in the circuit, the measured time with a timer, the known charging time, and battery voltage allow $R_{soil}$ to be determined.

The objectives of this research were (i) to assess sensor-to-sensor variability in measured resistance, (ii) to determine the temperature effects on sensor measurements at different salinity levels, and (iii) to evaluate the performance of the sensor under a wide range of soil water content and soil solution electrical conductivity for different soils in laboratory.

## 2. Materials and Methods

### 2.1. Sensor Description

The sensor (Sensoterra B.V.) is shown in Figure 1a; it has two prongs with different measurement depths (i.e., 15, 60, 30, and 90 cm), a battery, resistor, and IoT framework in the sensor overmold. The resistance is measured as a function of charge/discharge time duration with an oscillator supplying a square wave signal at a frequency of 70 MHz. This sensor has a maximum sampling volume of 6.2 cm^3^, with a resolution of approximately 0.1% across the sensor in the soil. The operational temperature range of this sensor is between −20 °C to +60 °C. The sensor uses the LoRaWAN communication protocol. LoRaWAN is low-power, wide-area networking protocol designed to wirelessly connect battery operated “things”, e.g., the Sensoterra sensor, to the internet. LoRaWAN network architecture is basically deployed in a star topology for a local area network (LAN), in which all gateways or nodes are individually connected to a central network server via standard IP connections and act as a transport bridge. The wireless communication uses the very long-range attributes of the LoRaO physical layer, allowing a single-hop link between the end device and one or even many gateways. The gateway can be placed at a distance up to 2.5 km from the sensors. The gateway uploads the data to the cloud and can be connected to many wireless soil moisture sensors. The data can be transmitted hourly and is downloaded from a web browser or via a mobile app in the form of an Excel file.

### 2.2. Assessing Sensor-to-Sensor Variability

Experiments were initially conducted using glass beads [26] and Oso Flaco sand [27] to assess the sensor-to-sensor variability in the form of soil resistance. The predetermined water content of 0.2 m^3^ m^−3^ and solution electrical conductivity of 1 dS/m were mixed with oven-dried soil. The soil was mixed by hand and immediately transferred to a plastic zip bag, kept in the darkroom, and occasionally shaken over 24 h to get evenly distributed water content. Later, the soil was packed in a calibration container (49 × 30 × 10 cm) layer-by-layer with careful compaction to ensure the soil was distributed evenly. The top of the container was covered with paraffin wax paper to minimize evaporation loss. Further, a total of 15 sensors were installed vertically into the soil in such a way that the sensor prongs were fully immersed into the soil. The sensors were installed carefully to avoid air gaps, and the sensor measurements were recorded for 24 h. Also, the room temperature was recorded every half-hour to evaluate the temperature effect on sensor readings. Statistical analysis was performed using the R statistical software version 3.6.2 [28] to assess the effect of sensor-to-sensor variability on sensors measurements.

### 2.3. Effect of Soil Type and Solution Electrical Conductivity on Sensor Performance

Soil-specific calibrations were performed using four types of soils viz. glass beads, Oso Flaco sand, Columbia loam, and Yolo clay loam. The physical properties of these four soil types are presented in Table 1. Twelve sensors were used for the soil-specific calibration with different water content and electrical conductivity solutions. Twelve sensors were used for calibration with glass beads, and these were reduced to six for the other three soil types. Undisturbed soil cores were collected for each soil type and processed to determine the bulk density and soil porosity, following standard procedures in [29]. The soil bulk density can be measured either by direct or indirect methods. Direct methods measure the mass and volume of oven-dried known volume of soil samples from sampling core, while indirect methods use the radiation or regression techniques to determine soil bulk density. In this study, we followed the direct method (core method) to measure the bulk densities of the soils used for sensor calibration [29]. Soil porosity can be easily obtained by using the relation between soil bulk density and particle density [30]. Required soil samples were air-dried for 24 h, and any unwanted materials, such as gravel and plant roots, were removed before passing through a 2-mm sieve [31]. A clear acrylic container (30 × 15 × 5 cm) was assembled to install the sensors at a depth of 3.5 cm so that the entire sensor measurement volume would be contained within the soil. The output of the sensor was the resistance (Ω) and average apparent volumetric water content collected at 30 min intervals.

The air-dried soil of known quantity was taken, and each sample was mixed with a known volume of deionized water and prerequisite electrical conductivity solution (made using KCl) to bring it to actual water content and soil solution electrical conductivity of 0.10, 0.20, 0.35, and 0.45 m^3^ m^−3^, and 0.5, 1.0, 1.5, 2.0, 3.0, 4.0, 5.0, and 10 dS/m, respectively. The soil was mixed thoroughly by hand to create a homogeneous mixture with designated actual water content. The mixture was then transferred to a calibration container in small increments while compressing the soil with a compaction hammer to compact the soil to the desired bulk density. After transferring the soil mixture into calibration containers, these were covered with paraffin wax paper to minimize the evaporation loss during the experiment. A total of six sensors were inserted vertically, similar to the procedure followed in assessing the sensor-to-sensor variability. The soil packing was repeated to prepare the desired water content and electrical conductivity solutions for all four soil types. After completion of each calibration experiment, the sensors were removed from the container, and the soil cores with known volume (70.5 cm^3^) were inserted into the soil centered around the sensor prongs. After weighing, the soil samples were dried for 24 h at 105 °C and then reweighed to determine the gravimetric water content. In total, four water contents and six electrical conductivity solutions for each soil type were used in this study, to observe a wide range of conditions.

A high precision TDR-315 sensor (Acclima) was used to assess the bulk electrical conductivity of each soil mixed with a known water solution. This was achieved by placing the Acclima sensor in the calibration container horizontally after sensor measurements. The measured bulk electrical conductivity (*EC_b_*) was related to actual water content (θ) following [32], as follows:

(8) $$EC_{b}=c_{1}EC_{w}\theta^{2}+c_{2}EC_{w}\theta +c_{3}$$

where $c_{1}$–$c_{3}$ are the regression coefficients, 𝑐_1_ and 𝑐_2_ are related to the soil’s tortuosity factors, and 𝑐_3_ is related to the surface conductance of the soil particles [33].

### 2.4. Assessing Sensor Sensitivity to Variations in Temperature

To determine the temperature effect on sensor measurements, experiments were conducted using glass beads as a reference medium. The air-dried soil samples of glass beads were mixed with predetermined amounts of water to obtain soil water contents of 0.1 to 0.45 m^3^ m^−3^ and about 0.5 and 1 dS/m electrical conductivity. These mixtures were then transferred into 1 L acrylic cylinders and covered with a paraffin wax paper to reduce the water content changes by evaporation before, and during, the temperature experiments. A constant temperature chamber (Forma Scientific, Marietta, OH, USA) was used in this experiment and was maintained at temperatures ranging from 5 to 45 °C, with an increase of 5 °C per hour during each experiment, as described in [23]. Temperature calibration was initiated by assuming the linear relation between apparent sensor resistance and temperature [31], which can be expressed as:

(9) $$\frac{d\Omega }{dT}=f\left(\theta \right)$$

where Ω is sensor apparent resistance, θ is actual water content, *T* is system temperature (°C), and f(θ) is a fitting function.

Integration of Equation (9) yields:

(10) $$\Omega =\Omega_{p,r}+f\left(\theta \right)\left(T-T_{r}\right)$$

where $\Omega_{p,r}$ represents the sensor read resistance at a reference temperature, and $T_{r}$ is room temperature.

To solve Equation (9) for Ω, first, empirical relations were developed between f(θ) and $\Omega_{p,r}$. Based on the coefficient of determination from different functions, i.e., linear, logarithmic, and exponential, we determined the best fit for the $\frac{d\Omega }{dT}$ vs. *θ* data for the sensor to be a third-order polynomial equation, as follows:

(11) $$f\left(\theta \right)=a_{0}+a_{1}\theta +a_{2}\theta^{2}+a_{3}\theta^{3}$$

where $a_{0}$ to $a_{3}$ are fitting coefficients. The value of $\Omega_{p,r}$ was approximated from the sensor, which provided default calibrated resistance values for sand instead of soil-specific calibration [34].

## 3. Results and Discussion

### 3.1. Sensor-to-Sensor Variability

**Figure 2:**
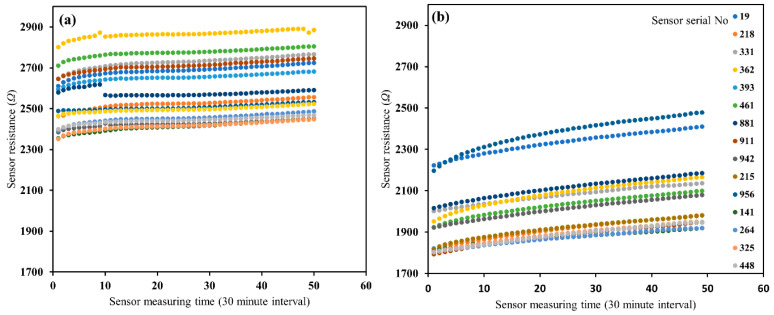
Comparison of 15 probes output resistance at 0.2 m^3^ m^−3^ water content with ~1 dS/m solution of electrical conductivity in (**a**) glass beads and (**b**) Oso Flaco sand, over 24 h.

**Figure 3:**
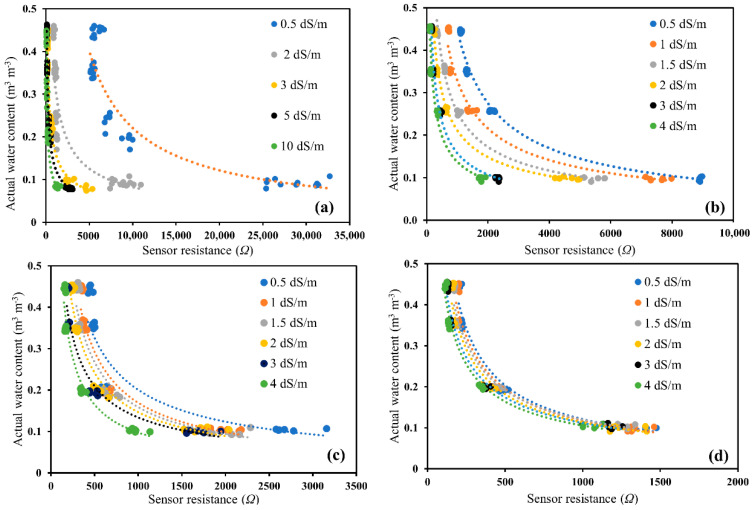
Changes in sensor output resistance (Ω) over a range of actual water content (volumetric water content) and solution electrical conductivity (*EC_w_*) in (**a**) glass beads, (**b**) Oso Flaco sand, (**c**) Columbia loam, and (**d**) Yolo clay loam.

The results of the sensor-to-sensor variability for 15 sensors, using glass beads and Oso Flaco sand, are shown in Figure 2. The statistical evaluation of sensor measurements using the coefficient of variation (*CV*) and ANOVA are presented in Table 2 and Table 3. A slight increase in resistance (Ω) with time was noticed, indicating either a change in temperature or a slight drying of the sandy soil with time. However, knowing that the temperature might have an impact on the sensor readings, the room temperature was found to vary between 23–23.5 °C over the duration of 24 h of the experiment. To quantify the variation among the sensors’ resistance (Ω) measurements during the soil-specific calibration, the *CV* was determined, which was defined as the standard deviation and mean ratio for each water content and solution salinity. It was observed that the variations in sensor resistance ranged from 1 to 30, 1 to 7, 2 to 10, and 1 to 8% for glass beads, Oso Flaco sand, Columbia loam, and Yolo clay loam, respectively (Table 2). The highest variation in glass beads was expected for lower water content and salinity ranges, as the resistance values were maximum in these ranges, and mostly nonlinear (Figure 3a).

The ANOVA test was carried out, wherein the total variance was divided into the variance between the sensors (sensor-to-sensor variability). The null hypothesis that all sensors have the same mean sensor response was verified by the F test [35]. The F value was higher than the critical F value of 1.71 (Table 3), which means the rejected null hypothesis indicated that at least one sensor had a significantly different mean sensor response. A high F value also indicates that the sensor-to-sensor variability is the dominant source of variation in the sensor response measurements. The variability of the 15 sensors was considerably higher. Therefore, the ANOVA test indicated that the mean sensor response was significantly different for at least one sensor (F value = 128 for glass beads and 600 for Oso Flaco in Table 3), indicating substantial sensor-to-sensor variability [35].

### 3.2. Effect of Soil Type and Solution Electrical Conductivity on Sensor Performance

Out of the 15 sensors used in the sensor-to-sensor variability experiments, only 12 sensors were selected to ease the experimental procedure to carry out soil-specific calibrations. Changes in sensor output resistance (Ω) were observed for all the sensors for four soil types having varying actual water content and electrical conductivity solutions, as shown through Figure 3a–d. For glass beads, the wide salinity range between 0 to 10 dS/m was used to detect the effect of salinity on the sensor resistance measurements, but the manufacturer indicated that the optimal working range of the sensor could be from 0.5 to 5 dS/m. For the other three soil types, the study was further conducted with salinity ranging from 0.5 to 4 dS/m [36]. The nonlinearity in the Ω–θ relationship varied with different soil types (Figure 3). The high nonlinearity of these calibration curves signifies that (i) with high water content, the sensor is highly sensitive but highly uncertain, and (ii) with low water content, the sensor is very insensitive with low uncertainty. This can be explained by the fact that water content has a strong impact on both the capacitive and conductive behaviors of soil, whereas salinity mainly affects the conductive behavior alone [37]. The salinity effect was more pronounced in sandy soils, such as glass beads and Oso Flaco, and was quite less in Columbia loam, and even more reduced in Yolo clay loam (Figure 3) than the other three soil types. It was observed that, going from sand to sandy loam to clay loam, the *EC_s_* (surface conductivity of soil particles) became more prominent, thus reducing the effect of solution electrical conductivity (*EC_w_*).

**Figure 4:**
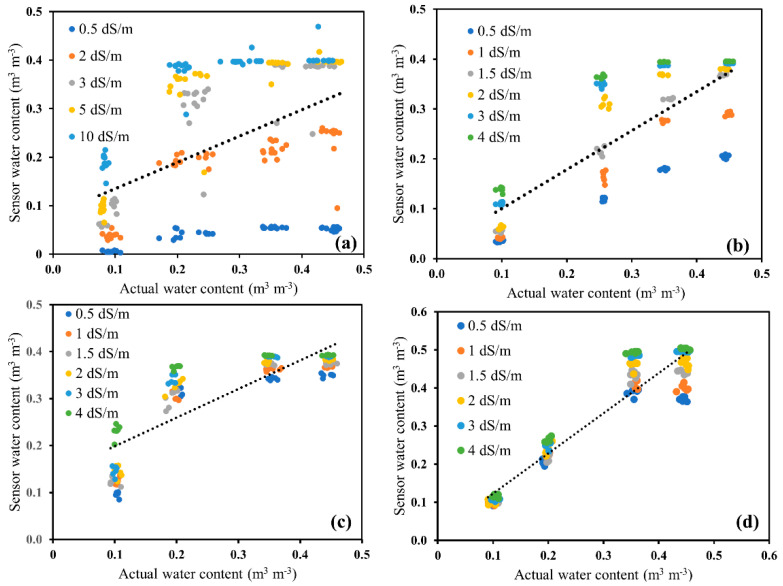
Comparison of combined sensor water content with gravimetrically-determined actual water content for (**a**) glass beads, (**b**) Oso Flaco sand, (**c**) Columbia loam, and (**d**) Yolo clay loam over a range of water and electrical conductivity solutions.

A regression analysis between the apparent water content measured by sensors and the actual water content is presented in Figure 4 for four soil types, which ranged from 0.5 to 10 dS/m solution salinity (*EC_w_*). It was observed that the data were highly scattered for glass beads when put together with all the water contents and salinity solutions, with an R^2^ of 0.25 and RMSE of 0.12. Similarly, the other three soil types had a correlation between observed and measured water contents of R^2^ ≥ 0.65 and RMSE, ranging from 0.05 to 0.09 (Table 4). However, the individual fitting for each electrical conductivity solution had a correlation above 0.95, which implied that using the calibration equation depends on each salinity level, instead of a single composite equation for all salinity levels [38]. Thus, we can conclude that soil-specific calibration is necessary for low-cost soil moisture sensors at each salinity level.

**Figure 5:**
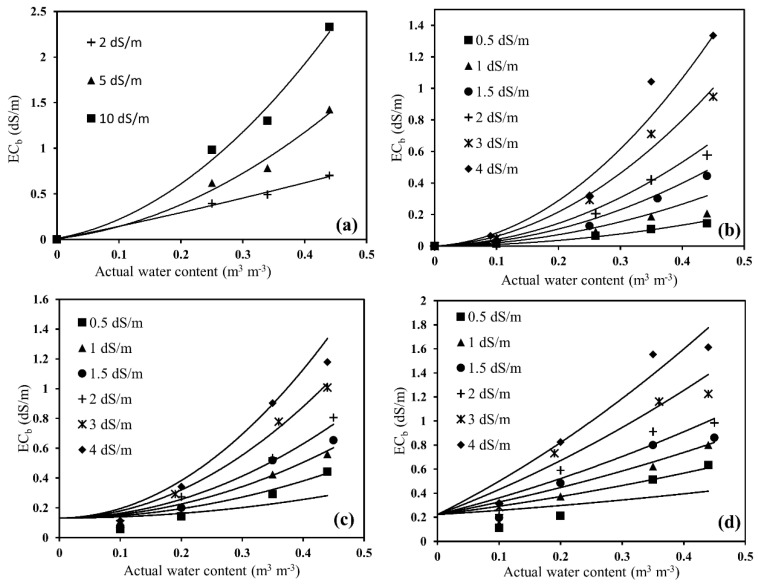
Calibration of sensor probe for measuring bulk electrical conductivity (*EC_b_*) as a function of water content and salinity in (**a**) glass beads, (**b**) Oso Flaco sand, (**c**) Columbia loam, and (**d**) Yolo clay loam (in Table 5, $c_{1}$, $c_{2}$ and $c_{3}$ are optimized parameters for the Rhoades equation [32]). Note that for both glass beads and sand, *EC_s_* can be assumed to be zero.

Soil bulk EC was measured for four soil types, and the data fitted to the Rhoades model [32] presented for the glass beads (Figure 5a), Oso Flaco sand (Figure 5b), Columbia loam (Figure 5c), and Yolo clay loam (Figure 5d). The regression coefficients are presented in Table 5. All the data points fitted well with the calibration model of Equation (6), with an R^2^ ≥ 0.90 and RMSE ranging from 0.051 to 0.121 dS/m for the four soil types indicating the variability between the sensor readings. Soil bulk EC was found to increase with actual water content and *EC_w_*, and regulated by the geometry of the surface conductivity of pores and soil particles. This was also revealed by the fitting functions presented through Figure 5a–d, with increasing values of *c*_3_, i.e., 0.0, 0.13, and 0.22 for Oso Falco sand, Columbia loam, and Yolo clay loam, respectively. Therefore, calibrations for sensor measurements in different solution salinity would be soil-specific [23].

### 3.3. Temperature Effect on Sensor Water Content Measurements

**Figure 6:**
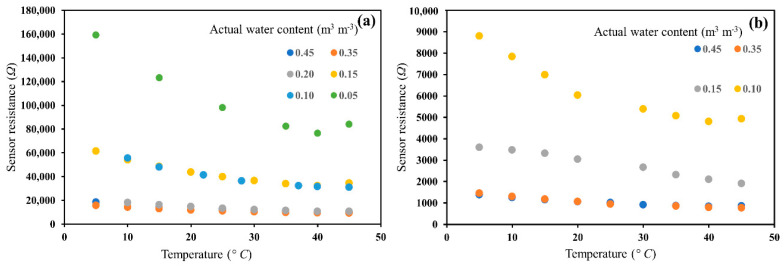
Response of the sensor to temperature at each actual water content and (**a**) at ~0.5 dS/m salinity, and (**b**) at 1 dS/m salinity in glass beads. Sensor data represents the raw data in resistance (Ω) at different temperatures.

**Figure 7:**
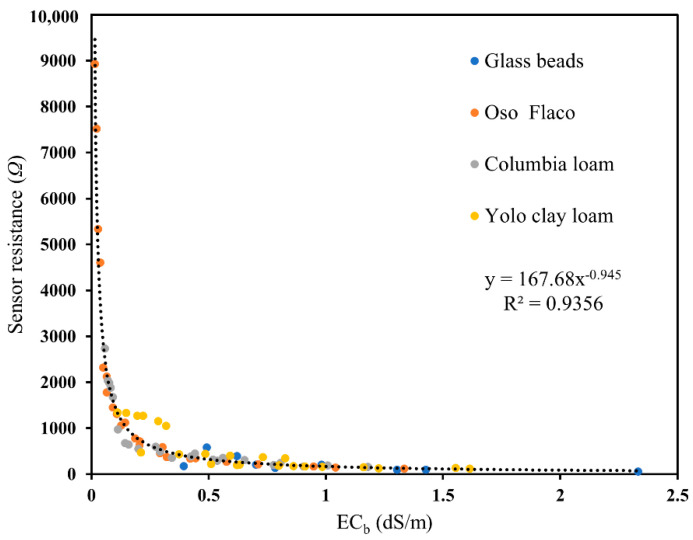
Dependence of soil bulk EC on sensor resistance (Ω) for all the four soil types used in this study.

The sensitivity of sensor measurements to temperature variations in glass beads for 0.5 and 1 dS/m salinity and a range of actual water contents are presented in Figure 6. The results show that the resistance decreased with temperature (Figure 6a,b) for both salinity levels. Also, the temperature effect became more apparent as water content or salinity decreased, corresponding to increasing resistance values. The sensor measurement is a direct resistance and is related to bulk soil EC, verified by the unique relationship between soil bulk EC and sensor resistance (Figure 7). Thus, the temperature effect can be corrected using the relationship described in [39].

**Figure 8:**
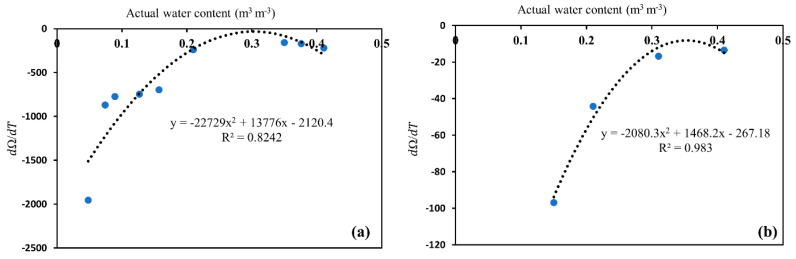
Dependence of dΩ/dT on water content at (**a**) 0.5 dS/m salinity, and (**b**) 1 dS/m salinity level. The dΩ/dT value is the slope of the linear response of sensor resistance to *T* in Figure 6.

The dΩ/dT curve showed a bidirectional relationship with increasing θ with sensitivity values of resistance ranging from 20 to 2500 Ω (Figure 8). The fitting coefficients were 0.82 for 0.5 dS/m and 0.98 for 1 dS/m salinity levels. These trends for the sensor were consistent with those reported by [31,34] for EC-5 and 5TE sensors as bidirectional patterns for different soils used to relate the apparent water content with temperature. However, the shape of the curve and the number of regression coefficients of sensor output and actual water content varied from the sensor type, irrespective of the trend, as shown by Equation (9) [34]. Therefore, this method appears to be appropriate for this type of sensor in a consistent format, irrespective of the form of their output- θ functions, provided that the dependencies of the slopes on θ can be fitted by some form of an empirical equation, as shown in Figure 8.

## 4. Conclusions

The performance of a low-cost wireless soil moisture sensor whose principle of operation is based on resistivity and capacitance was evaluated under four different soil types. Sensor-to-sensor variability experiments revealed significant variability between the sensors, expressed as coefficient variation (1 to 10%). The soil-specific calibration shows that the sensor exhibits nonlinearity in the Ω–θ relationship, which differs by soil type, indicating that the sensor is highly sensitive at higher water contents with high uncertainty, and insensitive at low water content with low uncertainty. The effect of salinity on sensor performance in four soil types was reduced from sand to sandy loam to clay loam, and the effect of *EC_s_* (surface conductivity of soil particles) became more pronounced, thus lowering the effect of solution EC (*EC_w_*). This was also witnessed by fitting the polynomial function between soil bulk EC and actual water content with increasing values of the surface conductivity of soil particles, which were reduced from 0.0 (sand), 0.13 (Columbia), to 0.22 (Yolo). Overall, we conclude that this low-cost wireless soil moisture sensor, after soil-specific calibration, can produce accurate estimates of soil water content in soils commonly found in agricultural fields, e.g., Yolo clay loam. It is worth noting that soil solution electrical conductivity and temperature can affect the sensor readings but can be corrected for using calibration curves similar to those developed in this study using laboratory observations of soil type and salinity level. The low cost and simple wireless communication protocol make this sensor suitable for precision irrigation and hydrologic high spatiotemporal monitoring of soil moisture.

## Figures and Tables

**Table 1:** Soil texture and effect of solution electrical conductivity (*EC_w_*) of soils used in calibration.

Soil	Sand	Silt	Clay	*EC_w_*	Dry Bulk Density
	%			dS/m	(g/cm^3^)
Glass beads	100	0	0	0	1.31 ± 0.02
Oso Flaco sand	100	0	0	0	1.35 ± 0.01
Columbia silt loam	54	30.9	15.1	0.41	1.25 ± 0.02
Yolo clay loam	32.4	36.8	30.8	0.48	1.26 ± 0.02

**Table 2:** Coefficient of variation (*CV*) of sensor resistance with varying actual water contents with respect to different electrical conductivity solutions (*CV* = *σ*/*μ* × 100%).

**Glass Beads**					**Oso Flaco Sand**				
**θ/EC**	**0.45**	**0.35**	**0.25**	**0.1**	**θ/EC**	**0.45**	**0.35**	**0.25**	**0.1**
**0**	4.9	12.4	10.9	8.5	**0.5**	2.2	1.3	3.5	0.4
**0.5**	8	2.5	15.2	8.8	**1**	2.1	1.2	7.4	3.9
**1**	1.4	7.9	6.4	12	**1.5**	2.7	0.9	3.7	5.4
**2**	2.9	3.9	14.2	32.1	**2**	2.1	1.7	5.9	6.5
**5**	8.4	6.2	18.5	9.8	**3**	4	1.4	5.5	2.2
**10**	14	24.2	18.1	11.8	**4**	1	3.7	4.4	4
**Columbia Loam**					**Yolo Clay Loam**				
**θ/EC**	**0.45**	**0.35**	**0.2**	**0.1**	**θ/EC**	**0.45**	**0.35**	**0.2**	**0.1**
**0.5**	3.81	2.27	6.27	7.31	**0.5**	3.09	1.77	6.32	7.21
**1**	2.54	3.61	6.08	7.59	**1**	2.59	3.14	4.39	4.48
**1.5**	4.34	3.68	10.57	8.06	**1.5**	1.22	3.85	8.05	3.28
**2**	5.45	6.31	10.07	10.22	**2**	3.08	3.19	6.73	7.73
**3**	3.75	3.34	9.04	8.11	**3**	1.99	1.86	4.22	6.79
**4**	4.18	2.87	6.24	7.61	**4**	4.83	2.61	2.92	4.67

**Table 3:** The ANOVA for 24 h of sensor response measurements (resistance) of 15 sensors (Figure 2) in the glass beads and Oso Flaco sand.

Source	Sum of Squares	df	Mean Square	F Value	*p*-Value	F Crit
Glass beads	73,879,231.21	15	4,925,282.08	128.089	0.000001	1.67915
Oso Flaco	18,616,279.45	15	1,329,734.24	600.183	0.0000001	1.70552

**Table 4:** Summary of combined probe calibration functions of the form: y = b0 + b1x.

Soil	b0	b1	R^2^	RMSE
Glass beads	0.081	0.543	0.25	0.124
Oso Flaco sand	0.022	0.781	0.65	0.091
Columbia silt loam	0.138	0.608	0.68	0.082
Yolo clay loam	0.017	1.053	0.89	0.058

**Table 5:** Optimized parameters for Rhoades model for all soils.

Soil	Regression Coefficients (dS/m)				RMSE (dS/m)
	$c_{1}$	$c_{2}$	$c_{3}$	R^2^	
Glass beads	0.89	0.15	-	0.96	0.051
Oso Flaco sand	1.52	0.06	-	0.97	0.063
Columbia silt loam	1.54	0.06	0.13	0.94	0.071
Yolo clay loam	0.56	0.64	0.22	0.90	0.121
